# Atrial Fibrillation Ablation: Current Practice and Future Perspectives

**DOI:** 10.3390/jcm12247556

**Published:** 2023-12-07

**Authors:** Laura Rottner, Andreas Metzner

**Affiliations:** University Heart and Vascular Center, University Hospital Hamburg-Eppendorf, 20251 Hamburg, Germany

**Keywords:** atrial fibrillation, catheter ablation, indication, patient selection, ablation technologies

## Abstract

Catheter ablation to perform pulmonary vein isolation (PVI) is established as a mainstay in rhythm control of atrial fibrillation (AF). The aim of this review is to provide an overview of current practice and future perspectives in AF ablation. The main clinical benefit of AF ablation is the reduction of arrhythmia-related symptoms and improvement of quality of life. Catheter ablation of AF is recommended, in general, as a second-line therapy for patients with symptomatic paroxysmal or persistent AF, who have failed or are intolerant to pharmacological therapy. In selected patients with heart failure and reduced left-ventricular fraction, catheter ablation was proven to reduce all-cause mortality. Also, optimal management of comorbidities can reduce AF recurrence after AF ablation; therefore, multimodal risk assessment and therapy are mandatory. To date, the primary ablation tool in widespread use is still single-tip catheter radiofrequency (RF) based ablation. Additionally, balloon-based pulmonary vein isolation (PVI) has gained prominence, especially due to its user-friendly nature and established safety and efficacy profile. So far, the cryoballoon (CB) is the most studied single-shot device. CB-based PVI is characterized by high efficiency, convincing success rates, and a beneficial safety profile. Recently, CB-PVI as a first-line therapy for AF was shown to be superior to pharmacological treatment in terms of efficacy and was shown to reduce progression from paroxysmal to persistent AF. In this context, CB-based PVI gains more and more importance as a first-line treatment choice. Non-thermal energy sources, namely pulsed-field ablation (PFA), have garnered attention due to their cardioselectivity. Although initially applied via a basket-like ablation tool, recent developments allow for point-by-point ablation, particularly with the advent of a novel lattice tip catheter.

## 1. Introduction

In the late 1990s, a discovery made by Haissaguerre and colleagues, which identified the electrical activity in the pulmonary veins (PVs) as the primary trigger for atrial fibrillation (AF) [1], prompted the electrophysiology community to abandon efforts to replicate surgical techniques developed by Cox and his team [2]. It has become evident that excitable tissue within the PVs plays a central role in the occurrence of AF. Consequently, the primary approach that emerged was to disrupt the electrical connections between the PVs and the left atrium (LA) via catheter ablation [3]. This strategy evolved from a segmental approach, targeting the earliest site of activation at the PV ostia, to an ablation strategy aiming at wide-area PV encircling. This was initially purely fluoroscopically guided, then later guided by an electroanatomic mapping system [4,5].

Pulmonary vein isolation (PVI) has demonstrated a success rate between 60% and 90% at one-year follow-up in patients with paroxysmal AF [6,7,8], while success rates in persistent or long-standing persistent AF are less favorable [9,10]. Catheter ablation for AF has been shown to be more effective with regard to maintenance of sinus rhythm when compared to pharmacological treatment [11], offers a significant improvement in quality of life [12], may delay or stop progression to more advanced forms of the disease [13,14], and has been proven to reduce all-cause mortality in selected patients [15]. Multiple energy sources and various ablation tools as well as different ablation strategies have been evaluated with regard to efficiency, efficacy, and safety [8,16]. With regard to arrhythmia-free survival and clinical outcome after AF ablation, patient selection, simultaneous risk factor management, as well as optimal time point for catheter ablation play a decisive role [17].

Within this review article, we summarize the current practice and future perspectives of catheter ablation of AF.

## 2. Recommendations for Catheter Ablation According to Current Guidelines

The main clinical benefit of AF ablation is the reduction in arrhythmia-related symptoms and therefore improvement of quality of life [12,17]. The results of the EAST AFNET-4 trial have shown that an early rhythm-control strategy is associated with lower rates of cardiovascular events when compared to usual care [18]. However, in the rhythm-control arm, the initial choice of therapy was flecainide (36%), followed by amiodarone (20%), dronedarone (17%) and only a minority of patients initially underwent catheter ablation (8%). So far, no randomized controlled trial has demonstrated a significant reduction in all-cause mortality with AF ablation in the “general” AF population. Therefore, the indications for AF ablation have not been broadened beyond symptom reduction, and AF ablation is not yet indicated in asymptomatic patients [17].

Catheter ablation of AF is recommended, in general, as a second-line therapy for patients with symptomatic paroxysmal or persistent AF, who have failed or are intolerant of class I or class III AAD therapy (class I) [17]. As an exception, in selected patients with heart failure and reduced left ventricular fraction (HFrEF), catheter ablation was proven to reduce all-cause mortality and re-hospitalization, and was also associated with significant improvement of left ventricular function [15,19,20]. Thus, the CASTLE-AF trial provides strong evidence and supports the current guideline recommendation to endorse catheter ablation as the preferred treatment of AF in patients with HFrEF, regardless of the type of AF (class I) [15,17].

Interventional cardiac procedures involve the potential risk of life-threatening complications [21]; however, several clinical trials considering AF ablation as a first-line therapy could demonstrate that catheter ablation is more effective at maintaining sinus rhythm when compared to AAD therapy [6,22,23]. Furthermore, long-term AAD therapy is known to be more commonly associated with considerable side effects compared to ablation (17% vs. 8%) [24]. Based on these findings and patient preferences, according to current guidelines, AF ablation should be considered before an AAD in patients with paroxysmal AF episodes (class IIa) or may be considered in patients with persistent AF without risk factors for recurrence (class IIb) [17].

## 3. Patient Selection and Optimal Timing for Atrial Fibrillation Ablation

There is compelling evidence indicating a strong association between the clinical presentation of AF and the outcomes following ablation. The rates of arrhythmia recurrence tend to increase progressively in patients with paroxysmal, persistent, and long-standing persistent AF. Consequently, the type of AF holds significant importance when assessing whether a patient should undergo AF ablation, and these considerations still align with the latest guideline recommendations. In addition, it is known that AF ablation may induce a reversal of atrial remodeling and recent studies also suggest that early ablation in the disease’s progression can prevent further deterioration. The prospective, controlled ATTEST trial, published in 2021, was conducted at 29 hospitals and medical centers across 13 countries and randomized paroxysmal AF patients in a 1:1 fashion to either radiofrequency (RF) ablation or AAD therapy. The primary endpoint was the rate of persistent AF/atrial tachycardia (AT) at 3 years of follow-up. The results of the study demonstrated that RF ablation is superior to guideline-directed AAD treatment in delaying the progression from paroxysmal to persistent AF. Recently, similar findings were published by Antrade et al. In their study, they report the three-year follow-up of patients with paroxysmal untreated AF who had been randomly assigned to undergo initial rhythm-control therapy with cryoballoon-based PVI or to receive AAD therapy. The authors found that initial treatment with cryoablation was associated with a lower incidence of recurrent atrial tachyarrhythmia and, of note, lower rates of persistent forms of AF over 3 years of follow-up when compared to pharmacological treatment. In this context, early catheter ablation—ideally in the early stages of the disease—with safe ablation tools is gaining more and more attention.

When weighing the risks and benefits of catheter ablation as an invasive procedure, it also makes sense to avoid ablation in patients who are considered to have a very low probability of success due to significant and unmodifiable risk factors. However, it is important to determine whether some of these patients could benefit from ablation despite lower arrhythmia-free survival rates, as has already been shown, for example, for patients with heart failure [15]. Recently, results of the CASTLE-HTx randomized trial have demonstrated that even among AF patients with end-stage heart failure, the combination of catheter ablation and guideline-directed medical therapy was associated with a lower likelihood of a composite of death from any cause, implantation of a left ventricular assist device, or urgent heart transplantation than medical therapy alone, which might be due more to relevant reduction in the AF burden and a return from persistent to paroxysmal episodes as due to complete arrhythmia-freedom [25]. In addition, data from the EAST AFNET-4 subanalysis already support an early rhythm-control strategy in patients with a high comorbidity burden [26]. While there are no prospective data investigating whether this is not only for the early rhythm-control approach in general but also specifically for catheter ablation, current knowledge suggests that a holistic therapy concept certainly plays a crucial role in this patient population, which includes not only effective rhythm-control by catheter ablation but also the treatment of cardiovascular risk factors [17]. Already older observational data have shown that risk factor management can reduce the risk of AF recurrence after AF ablation to rates comparable to those of low-risk patients. In the ARREST-AF trial, published in 2014, risk factor management, including treatment of arterial hypertension, lipids, diabetes, sleep apnea, reduction of smoking and alcohol consumption, or weight loss was offered to AF patients undergoing ablation. After a median follow-up of 3.5 years, risk factor management led to a significant increase of arrhythmia-free survival compared to controls. Thus, at present, current guidelines recommend that for a more balanced indication for ablation in AF patients with risk factors for recurrence, the most intensely evaluated risk predictors (including total AF duration) should be considered, and adjusted to their preferences [17].

## 4. Ablation Strategies: First Step Pulmonary Vein Isolation

Since Haissaguerre et al. published a study in 1998, which revealed that PV triggers are the most important initiators of AF, electrical isolation of the PVs using catheter ablation has become the cornerstone for the treatment of affected patients. PVI has demonstrated a success rate between 60 to 90% at one-year follow-up in patients with paroxysmal AF [6,7,8]. Results after a single ablation procedure in patients with persistent and long-standing persistent AF are sobering; only about 51% of the treated patients remain free of arrhythmia-recurrence after one year and only 42% after three years of follow-up. After several interventions, success in this population is more promising at long-term follow-up at almost 69%; however, in the underlying meta-analysis only a few studies reported results of ablation after more than three years [27].

The main mechanism for the recurrence of AF after primary successful PVI is the reconnection of previously isolated PVs [28,29]. Lin et al. found that the majority of patients with AF recurrence who underwent re-ablation procedure after a mean follow-up of 36 months had reconnected PVs [30].

However, PVs might not be the only contributors to the intricate process of AF initiation and recurrence, and some patients may present recurrences despite durable PVI (“PVI non-responders”). External triggers beyond the PVs, as well as the disposition of interstitial fibrosus tissue and autonomic innervation as well as genetic predisposition, are likely to be co-factors in AF occurrence. However, the extent of their involvement in AF patients remains not fully comprehended and may vary from one patient to another.

In patients suffering from persistent and long-standing persistent AF, due to disappointing ablation success rates, and in the context of so-called “PVI non-responders”, more extensive ablation beyond PVI has been advocated. This includes linear lesions, isolation of the left atrial appendage (LAA) or of the superior vena cava, ablation of complex fractionated atrial electrograms (CFAE), rotors, non-pulmonary foci, or ganglionated plexi, fibrosis-guided voltage and/or MRI mapping. However, research on ablation strategies beyond PVI, both during the index procedure [31,32,33] and during re-procedures with durable PVI [34,35], has not consistently demonstrated superiority with regard to recurrence rate in both, paroxysmal and persistent AF.

The prospective and randomized Alster-Lost AF trial assessed the outcomes of two index ablation strategies: standalone PVI versus a stepwise approach involving PVI followed by complex fractionated atrial electrogram (CFAE) ablation and the establishment of linear lesions in patients with symptomatic persistent or long-standing persistent AF. After 12 months, there were no significant differences in terms of freedom from atrial tachyarrhythmias between both groups [32]. Similarly, the CHASE-AF clinical trial compared the arrhythmia-free survival between standalone PVI and a stepwise approach, which included CFAE ablation and additional linear lesion creation in patients with persistent AF, and also revealed no advantage for the stepwise approach [33]. Lastly, the multicenter STAR-AF 2 trial compared PVI only and either PVI plus CFAE ablation or PVI plus creation of left atrial (LA) linear lesions in patients suffering from persistent AF. The results of STAR-AF 2 were in line with previous studies and could not demonstrate the benefit of more complex ablation strategies [31]. Consequently, PVI remains the only reproducible endpoint of AF ablation so far.

PVI can be accomplished using either a point-by-point or a single-shot technique. For the former approach, the operator performs a point-by-point wide-area circumferential ablation (WACA) line around the ipsilateral PV antrum using a single-tip catheter in combination with a 3D mapping system in order to achieve PVI. In contrast, when using a single-shot device, PVI is achieved with a single ablation application for each of the PVs [8,36]. Furthermore, different energy sources can be used for ablation itself. Briefly, we can divide energy sources into thermal energy (RF, cryoablation, laser) and non-thermal energy (electroporation/pulsed-field ablation).

### 4.1. The Classic in the Context of AF Ablation: Radiofrequency-Based Point-by-Point PVI

RF was the first energy source employed for AF ablation and is still the most used worldwide. RF ablation combined with an electro-anatomical mapping system allows a significant reduction in radiation exposure [37], provides additional information, e.g., the LA activation pattern and LA voltage, and therefore enables for additional (non-PV trigger) ablation as well as ablation of atrial tachycardias, if needed. However, this technique is intricate and involves a long learning curve, and even for experienced operators it still remains challenging to achieve contiguous, transmural, and permanent lesions using RF-based single-tip ablation [28,38].

Over time, there has been significant progress in enhancing RF catheter technologies with the goal of creating a uniform ablation lesion set while maintaining safety. A pivotal development in this direction was the introduction of irrigated-tip catheters. Over two decades ago, early investigations focused on PVI using single-tip, non-irrigated catheters, and RF delivery in a temperature-controlled mode [39]. Since the introduction of irrigated catheters, reducing thrombus formation and therefore allowing more power delivery, RF ablation is now predominantly performed in a power-controlled mode. Usually, RF ablation using irrigated-tip catheters involves applying moderate power (20–40 W) for a duration of 20–60 s. The quantity of energy delivered, as well as the level of catheter stability and tissue contact, are the primary factors influencing the formation of enduring lesions. The introduction of an ablation catheter equipped with contact force (CF) sensing capabilities has enabled real-time assessment of both catheter contact with atrial tissue and catheter stability. Several clinical studies have affirmed that a low CF during RF application is associated with acute procedural failure and sites of PV reconnection [40,41]. The reported one-year arrhythmia-free survival in patients with paroxysmal and persistent AF who underwent CF-guided RF-ablation ranges from 50–90%, respectively [42,43]. The question of whether CF-guided AF ablation leads to favorable clinical results especially with regard to improved safety cannot be clearly answered [44]. Furthermore, there is ongoing speculation on whether AF ablation using CF-sensing catheters might be associated with a higher incidence of atrioesophageal fistula formation, although similar overall complication rates have been found [45,46]. CF-sending further enabled the development of algorithms aiming at real-time assessment of lesion quality including the force–time integral (FT) [47], lesion size index (LSI) [48], and ablation index (AI) [49]. When coupled with automatic tagging and standardized workflows, aiming for contiguous lesions, the integration of lesion indices improved outcomes [50]. Moreover, Philips and colleagues conducted a comparison between CF-guided ablation protocol that utilized region-specific criteria of lesion contiguity and lesion depth (“CLOSE” protocol) and conventional PVI, and demonstrated favorable 1-year-clinical outcomes for patients suffering from paroxysmal AF [51].

In recent times, there have been additional advancements in RF-ablation with a particular focus on a strategy known as “High Power Short Duration” (HPSD). This approach aims to further enhance lesion quality and decrease ablation and procedure time. Contrary to common concerns, findings from ex vivo and in vivo animal studies consistently support the idea that HPSD ablation results in broader but more shallow lesions [52,53]. Thus, this technique may help to prevent damage to adjacent anatomical structures, such as the esophagus, during ablation procedures. Multiple clinical investigations, including a pair of randomized trials, demonstrated that contiguous, index-based encirclement with high power ablation in power-controlled mode does shorten the procedure time while maintaining a safe and effective procedure profile [54,55,56].

To address the limitation of reduced accuracy in tissue temperature feedback with conventional irrigated-tip catheters, innovative catheters equipped with multiple thermocouples have been developed, enabling more precise and real-time monitoring of tissue temperature. The novel DiamondTemp^TM^ ablation system (Medtronic^®^, Inc., Minneapolis, MN, USA) was designed to revive the advantages of temperature-controlled RF delivery. The tip of the DiamondTemp^TM^ ablation catheter incorporates six externally located thermocouples and a network of industrial diamonds to shunt heat from the catheter tip, allowing for precise temperature monitoring and low irrigation flow rates. The split-tip electrode provides real-time high-resolution electrograms and impedance recordings. In the randomized Diamond AF study, the DiamondTemp^TM^ ablation system demonstrated non-inferiority compared to standard CF-guided ablation, achieving higher overall power delivery and reduced procedure times [57]. Also, in a real-world cohort, the DiamondTemp^TM^ ablation system demonstrated high efficacy for PVI [58]. Likewise, a recent study demonstrated that the CF-sensing QDOT catheter (Biosense Webster, Irvine, CA, USA), together with temperature-controlled ablation up to 90 W during low-flow irrigation, enables high first-pass success rates for PVI while maintaining a balanced safety and effectiveness profile [59].

In conclusion, over the past 25 years, point-by-point RF ablation has evolved into a clinically proven, safe, and effective procedure due to standardization and ongoing innovation. Utilizing HPSD, whether in power- or temperature-controlled mode, has made it possible to fasten the procedure and therefore improve efficiency, all while maintaining safety and efficacy.

### 4.2. Step by Step to a Second Gold Standard: Cryoballoon-Based PVI

Apart from 3D electroanatomical mapping-guided RF-based ablation, cryoballoon (CB) ablation has emerged as an established gold standard for PVI [17]. Since its introduction into clinical practice, far more than one million patients worldwide suffering from symptomatic AF have been treated with different versions of this first single-shot device. There is a great amount of data on the efficacy and safety of the CB, and the CB is therefore, so far, the best studied single-shot ablation technology. CB-based PVI is characterized by high procedural reproducibility [60] and therefore high efficiency, convincing acute, mid-, and long-term success rates [8,61,62], and a beneficial safety profile combined with short learning curves [63,64,65].

Up until 2016, CB-PVI was considered a competitor to RF current in catheter ablation of AF. However, the FIRE and ICE trial, which enrolled patients with symptomatic paroxysmal AF and randomly assigned them to either RF- or CB-based PVI, established the CB as an equally effective and safe ablation technology [8]. It is worth noting that some may argue that the FIRE and ICE trial’s results may not fully represent real-world scenarios, as it was conducted in highly experienced electrophysiology (EP) centers. Nevertheless, the FREEZE cohort study involved 44 centers and included over 4000 patients suffering from paroxysmal and persistent AF, and could confirm that CB ablation was non-inferior to RF-based ablation in terms of both efficacy and safety [66].

The effectiveness of CB ablation in treating paroxysmal AF ranges from 65–80% at one-year follow-up [6,7,8]. Recently, it has been found that CB PVI, compared to pharmacological therapy, reduces the progression of paroxysmal AF toward persistent forms of AF [14]. As already discussed, so far, ablation strategies extending beyond pure PVI have not consistently demonstrated superiority over PVI-only approaches. Thus, PVI remains the only established, reproducible, and therefore recommended endpoint in first ablation procedures for AF, even in patients with persistent AF. Notably, there are rare data from randomized trials comparing CB ablation to classic RF-based AF ablation in subjects suffering from persistent AF. However, in the multicenter CRYO4PERSISTENT AF trial, CB PVI for the treatment of persistent AF demonstrated a 61% single-procedure success at 12 months post-ablation. In this study, compared with baseline recordings, there were also significantly fewer patients with arrhythmia-related symptoms at 12 months (16% vs. 92%; *p* < 0.0001). The symptom reduction was supported by significant improvement in 36-Item Short Form Health Survey composite scores and European Heart Rhythm Association score at 12 months [67]. The FIRE and ICE II randomized outcome trial has also been specifically designed to assess the effectiveness and safety of PVI using the CB versus RF energy in patients with persistent AF [68]. The results of this trial are eagerly anticipated.

Data on the safety of CB-based AF ablation report an incidence of major complications ranging from 2.0 to 7.5% [69,70]. Of note, the incidence of cardiac tamponade at CB-based PVI has been previously reported to be considerably low with 0.2 to 0.6% [71], and CB ablation, in general, is associated with lower risk of pericardial effusion and tamponade when compared to RF-based AF ablation [64]. The low incidence of cardiac tamponade during CB ablation may be mainly attributed to the over-the-wire technique and due to the lack of overheating and therefore steam pop risk. On the other hand, PNP has proven to be the most frequently observed complication in CB-based ablation, and in the literature, the clinical occurrence rate of PNP during CB procedures is reported from 1.7% up to 19.5% [65,70,72,73]. However, most PNPs are asymptomatic and transient [8,67].

In accordance with current AF guidelines, a history of ineffective or non-tolerated pharmacological treatment has traditionally been considered a prerequisite for catheter ablation. However, as already mentioned, early rhythm control has been shown to be favorable for reducing cardiovascular events in the main cohort of the EAST-AFNET 4 trial and subgroup analyses [18,26]. Therefore, the most effective rhythm-control strategy, which is catheter ablation, is gaining more and more importance [74] and within this context, CB-PVI plays a major role as a first-line ablation tool. Three recent randomized controlled studies suggest that adopting a first-line ablation approach for newly diagnosed AF using the CB is not only more effective than AAD therapy but also demonstrated non-inferior safety [6,22,23]. However, whether first-line therapy for AF using the CB or other single-shot devices can be implemented for the general AF population certainly also depends on healthcare economics and infrastructural factors, which should desirably also play a role in future analyses on this topic.

### 4.3. The Revolutionary Ablation Technology: Pulsed Field Ablation

Pulsed field (PFA) is a promising technology and PFA ablation is rapidly gaining importance in the field of AF-ablation. PFA ablation is a predominantly non-thermal energy source that involves exposing cardiac tissue to a brief yet intense electrical field. This leads to the creation of irreversible pores in the lipid bilayer (electroporation) and subsequent cell death [75]. The threshold for achieving irreversible electroporation varies depending on the type of tissue, and it is relatively low for cardiomyocytes when compared to nearby structures [76]. PFA’s tissue selectivity was confirmed in pre-clinical and clinical studies showing low vulnerability of nerves, vasculature, and esophageal tissue to PFA [77,78]. Literature indicates that, for equivalent lesion depths, PFA lesions are wider, larger, and more symmetrical than RF lesions, and PFA may be advantageous over RF ablation for the ablation of heterogeneous scar tissue [79,80].

Several catheters with varying designs (single-shot, basket-like, focal tip, and lattice catheter) are currently in different stages of development and clinical testing. The initially launched FARAPULSE catheter (Boston Scientific, Marlborough, MA, USA) incorporating a basket and flower-like catheter design was evaluated in several international multicenter surveys and trials [81,82,83].

In the MANIFEST international multicenter survey, acute procedural success involving the isolation of the PVs was almost universally achieved. Complications were rare and mainly not associated directly with PFA. The results of a multicenter registry by Schmidt et al. [83] and other single-center studies supported these findings [84,85]. Within the PersAFOne single-arm study, evaluating bipolar PFA using a multispline catheter for PVI and LA posterior wall ablation, invasive remapping at 2–3 months after the index procedure, confirmed the high durability of previously applied lesions [86]. Whereas initial reports focused on data without control groups, the recently published ADVENT trial compared thermal energy sources (RF and Cryoablation) versus PFA [81]. Within the ADVENT trial among patients with paroxysmal AF undergoing catheter ablation, PFA was non-inferior to conventional thermal ablation with respect to freedom from a composite of initial procedural failure, documented atrial tachyarrhythmia after a 3-month blanking period, AAD usage, cardioversion, or repeat ablation, and with respect to device- and procedure-related serious adverse events at 1 year [81].

Overall, previous literature shows reproducible data with regard to efficacy, durability, and safety when using PFA for catheter ablation of AF. In general, PFA can lead to typical complications associated with AF ablation, such as vascular complications, pericardial tamponades, and thromboembolic events. However, due to its cardioselectivity, injuries to surrounding structures—including phrenic nerve palsy or atrioesophageal fistula, one of the most feared and lethal complications during AF ablation [87]—appear to occur less or not at all [82,83].

The multispline-catheter design of the FARAPULSE catheter (Boston Scientific) allows straightforward isolation of the PVs. Successful additional substrate modification for AF via isolation of the posterior wall was reported [86,88]. However, PFA-capable multispline catheters lack the ability to deliver individualized, focal lesions with different energy levels and therefore certain limitations existed targeting other tachycardias. This called for PFA technology which is embedded in a 3D mapping platform allowing for the creation of highly accurate high-density maps in conjunction with the opportunity to draw distinct ablation lines with a single-tip catheter. Recently, a novel PFA system (CENTAURI; Galvanize Therapeutics, Phoenix, AZ, USA) was introduced, which enables monopolar biphasic PFA with commercially available, CF-sensing focal catheters in combination with their associated 3D mapping system. First single-center experiences reported convincing results during point-by-point PVI using this system [89]. However, besides PVI, effective and safe ablation of other structures, critical for atrial arrhythmias, such as the left atrial mitral isthmus (LAI) might be targeted during a left atrial ablation procedure. However, limited experience exists for PFA applications in the area of the LAI. Besides a limited effect of PFA in LA areas with thicker myocardium, complications such as coronary spasms or narrowing have been observed in select patients [86,90]. Thus, an ablation system enabling both PFA and a conventional thermal energy source such as RF was desirable for combining high flexibility and safety for the ablation of atrial tachycardias. Lately, a novel mapping and ablation platform (Affera^TM^, Medtronic, Singapore) combined with a compressible lattice-tip catheter (Sphere-9^TM^, Medtronic) was introduced, enabling focal ablation using either RF or PF energy [91]. After convincing animal and preclinical studies [92], the first in-person investigation could confirm that AF ablation with this focal RF/PF catheter allows efficient procedures, chronic lesion durability, and satisfactory freedom from atrial arrhythmias [93]. Further investigations of this innovative mapping and ablation tool are eagerly awaited.

## 5. Conclusions

Catheter ablation has become the leading therapeutic strategy for rhythm control in AF patients. Advances in technologies have led to increasing improvements in efficiency, effectiveness, and safety. Continuous advancements and emerging evidence are broadening the potential applications of catheter ablation as the primary treatment choice for AF, potentially even for asymptomatic patients. However, despite improvements in ablations techniques, the long-term outcomes following a single procedure are still not ideal for patients with persistent and long-standing persistent AF. Although, according to current literature, PVI remains the first and fundamental ablation strategy for all types of AF, further investigations are necessary to identify alternative strategies when PVI alone proves to be insufficient.

## Data Availability

Data are contained within the article.
